# Foot Temperature by Infrared Thermography in Patients with Peripheral Artery Disease before and after Structured Home-Based Exercise: A Gender-Based Observational Study

**DOI:** 10.3390/jpm13091312

**Published:** 2023-08-27

**Authors:** Anna Crepaldi, Lorenzo Caruso, Giovanni Piva, Luca Traina, Vincenzo Gasbarro, Roberto Manfredini, Nicola Lamberti, Natascia Rinaldo, Fabio Manfredini, Pablo Jesus Lopez-Soto

**Affiliations:** 1 Department of Nursing, Instituto Maimónides de Investigación Biomédica de Córdoba, 14004 Cordoba, Spain; anna.crepaldi@edu.unife.it (A.C.); pablo.lopez@imibic.org (P.J.L.-S.); 2 Department of Nursing, Pharmacology and Physiotherapy, Universidad de Córdoba, 14004 Cordoba, Spain; 3 Department of Nursing, Hospital Universitario Reina Sofía de Córdoba, 14004 Cordoba, Spain; 4 Department of Environmental and Prevention Sciences, University of Ferrara, 44121 Ferrara, Italy; lorenzo.caruso@unife.it; 5 Department of Humanities, University of Ferrara, 44121 Ferrara, Italy; giovanni.piva@unife.it; 6 Unit of Vascular and Endovascular Surgery, University Hospital of Ferrara, 44124 Ferrara, Italy; l.traina@ospfe.it (L.T.); vincenzo.gasbarro@unife.it (V.G.); 7 Department of Medical Sciences, University of Ferrara, 44124 Ferrara, Italy; roberto.manfredini@unife.it; 8 University Center for Studies on Gender Medicine, University of Ferrara, 44121 Ferrara, Italy; 9 Department of Neuroscience and Rehabilitation, University of Ferrara, 44121 Ferrara, Italy; nicola.lamberti@unife.it (N.L.); natascia.rinaldo@unife.it (N.R.); 10 Program of Vascular Rehabilitation and Exercise Medicine, University Hospital of Ferrara, 44124 Ferrara, Italy

**Keywords:** peripheral artery disease, infrared thermography, noninvasive, exercise, gender differences, claudication, exercise testing, exercise therapy

## Abstract

Decreased arterial perfusion is a typical condition of patients with peripheral artery disease (PAD), with the microvascular picture particularly present among women. This observational study aimed to detect foot perfusion changes by infrared thermography (IRT) after a home-based exercise program in both sexes. A total of 76 PAD patients with claudication (72 ± 4 years; 52 males) were enrolled in a structured in-home exercise program composed of two daily 8 min interval walking sessions (1:1 walk:rest ratio) with progressively increasing speed. Outcome measures collected at baseline (T0) and at each hospital visit after 5 weeks, 12 weeks and 20 weeks included foot temperature measured by IRT (anterior tibial, posterior tibial, dorsalis pedis and arcuate artery regions), ankle brachial index and the 6 min walking test. After 20 weeks, foot temperature in both limbs showed a significant increasing trend, with a mean variation of 1.3 °C for the more impaired limb and 0.9 °C for the contralateral limb (t = 8.88, *p* < 0.001 and t = 5.36; *p* < 0.001, respectively), with significant changes occurring after 5 weeks of training. The sex-oriented analysis did not highlight any significant difference, with an improvement of mean foot temperature of 1.5 ± 0.6 °C in females versus 1.2 ± 0.5 °C in males (*p* = 0.42). Ankle brachial index and performance also significantly improved over time (*p* < 0.001) without gender differences. In patients with PAD, a structured low-intensity exercise program significantly improved foot temperature and exercise capacity without any sex-related difference.

## 1. Introduction

Peripheral artery disease (PAD) is a frequent and common pathology that affects millions of people worldwide, particularly starting in the sixth decade of life [1]. Traditional risk factors such as smoking, hypertension, hyperlipidemia, diabetes and chronic kidney disease are mainly responsible for atherosclerosis of the arteries, including in the lower limbs [2]. In particular, smoking is associated with PAD, with increased risk occurring with higher smoking intensity and with a population-attributable fraction estimated at 44% [2,3]. The association between PAD and smoking persists after smoking cessation, although it is considerably diminished >10 years after cessation [2]. Hypertension is associated with an increased prevalence of PAD (odds ratio: 1.32 to 2.20), in particular among older patients, with a 20 mmHg increase in systolic blood pressure found to be associated with a 63% higher risk for PAD [2,4]. A high prevalence of hypercholesterolaemia is a significant contributor to PAD [2]. In most studies, total cholesterol has been strongly and independently associated with prevalent PAD [2,3,5]. Diabetes is strongly associated with PAD, with risk of developing the disease increased from 1.9 to 4.0 [5]. Diabetes also leads to poorer prognosis for PAD patients when compared to non-diabetic patients, with fivefold increased risk of amputation in relation to a specific pattern more frequently affecting distal arteries, frequent coexistence of neuropathy and higher risk of infection [2,6]. Finally, several markers of inflammation (e.g., high-sensitivity C-reactive protein, fibrinogen and interleukin-6) were found to be associated with PAD presence and progression, as well as some autoimmune diseases [2,7,8]. The management and control of traditional risk factors is mandatory at all stages of the disease [1,2].

In the intermediate stages of PAD, reduced oxygen delivery in the lower limb muscles results in ischemia-induced leg fatigue or discomfort while walking, with symptoms relieved by rest. In the more advanced stages, PAD may be responsible for critical rest pain or foot ulcers [1,9]. The evaluation of a PAD patient begins with their clinical history, a review of symptoms and physical examination by pulse palpation [1]. To confirm the diagnosis of PAD, abnormal physical examination findings must be confirmed with diagnostic testing, generally with the ankle brachial index (ABI) as an initial test, followed by duplex ultrasound, computed tomography angiography, magnetic resonance angiography or invasive angiography [1]. Moreover, to selectively determine foot perfusion, other specific methods can be applied, including the toe brachial index, transcutaneous oxygen pressure or skin perfusion pressure measurements or near-infrared spectroscopy (NIRS)-assisted tests [1,10,11]. All these techniques, in some cases classified with a IIa recommendation by the guidelines, present strengths and weaknesses [1]. Recently, infrared thermography (IRT) has emerged as a promising imaging method due to its capacity to detect the infrared light emitted by the body and visualize body temperature changes related to blood flow [12,13]. This noncontact, noninvasive and low-cost technique allows for a rapid evaluation of the energy radiation from the patient’s body. Due to its characteristics, IRT is safe for patients and healthcare professionals, and it is reliable for the measurement of skin temperature [13,14,15].

As highlighted in a recent systematic review [13], IRT has been employed to diagnose PAD, to evaluate the correlation between foot temperature and ABI [13], to assess the effectiveness of revascularization techniques and to evaluate the risk of negative outcomes for ischemic feet [16,17]. From this perspective, considering that the prevalence of so-called small vessel disease, a group of microvascular diseases with various etiologies affecting the small arteries, arterioles, venules and capillaries, has been found to be higher in women than in men [18], the topic may be of specific sex-related interest.

To the best of our knowledge, no manuscript has addressed variations in foot perfusion after exercise programs. In the intermediate stages of the disease, exercise therapy acts as a first-line option in PAD patients and has proven effective in improving symptoms and quality of life [1,2]. Different kinds of exercise programs have been studied throughout the years. Supervised exercise therapy requires patients to visit the hospital at least three times per week for a period ranging from 12 to 24 weeks. During each training session, the patient is asked to walk, usually on a treadmill, for at least 30 to 50 min at an intensity high enough to induce claudication pain. When the pain is unbearable, the patient is allowed to stop, restarting walking as soon as possible. Training progression is promoted throughout the sessions by increasing the walking speed or walking time. A median increase in walking distance of almost 180 m has been reported after supervised exercise programs [2,9]. Supervised exercise training is safe, and routine cardiac screening beforehand is not required, although such interventions are not reimbursed or available everywhere [2]. On the other hand, home-based programs were developed to overcome some of the barriers typical of structured exercise therapy. Several different protocols have been proposed in the literature, with or without the support of tracking devices, but the training program is similar to those developed in supervised sessions, with the patient asked to walk around the block at his/her place, facing and enduring claudication pain for at least 30 min at least five times per week [1,2,9]. Another training option is represented by a structured home-based program that prescribes precise walking and resting times to each patient, as well as prescriptions for walking speed and the total number of exercise bouts to be performed. The test-in–train-out program employed in this paper belongs to this category and is described detail later. Although home-based walking is not as effective as supervised exercise, it is a useful alternative, with positive effects on quality of life and functional walking capacity [2]. 

As highlighted in a recent Cochrane review, supervised exercise programs are not associated with ABI improvements [19], unlike a structured home-based program, with significant improvements in ABI observed after interval low- to moderate-intensity walking [20,21]. Nevertheless, changes in lower limb perfusion would represent a favorable outcome following exercise programs in PAD patients, with the possibility of improving their walking ability through increased oxygen delivery and impacting the rate of revascularizations or amputations [17,22]. 

This observational study aims to determine if changes in foot temperature measured by IRT in PAD patients may occur after completing a structured home-based exercise program, whether these changes correlate with ABI variation and whether sex differences may be observed.

## 2. Materials and Methods

### 2.1. Study Design and Setting

This observational prospective study was conducted at the Unit of Rehabilitation Medicine at the University Hospital of Ferrara between January 2020 and November 2022. The Ethics Committee of CE-AVEC approved the study. The study is reported in accordance with the STROBE guidelines.

### 2.2. Subjects

For this study, all consecutive patients with PAD between January 2020 and June 2022 screened at the Unit of Vascular Surgery, then referred to the Unit of Rehabilitation Medicine for the exercise program were evaluated for eligibility. The inclusion criteria were as follows: age > 60 years old and PAD at Leriche–Fontaine stage II. Patients were excluded from the analysis if they presented an ABI value > 1.41 or if they demonstrated the presence of incompressible vessels, chronic CEAP class III venous insufficiency, high or moderate to severe lower limb edema, severe cognitive impairment, absolute contraindication of exercise training (e.g., heart failure at NYHA class III or higher) or a very good exercise capacity (six-minute walking distance (6MWD) > 500 m).

### 2.3. Interventions

All patients were referred to an in-home structured exercise program named the TiTo-SHB, the details of which are reported elsewhere [23]. This structured walking program is prescribed at the hospital and includes two daily 10 min sessions of intermittent walking (walk:rest ratio of 1:1). The initial walking speed is approximately 50–60% of the habitual walking speed and progressively increases over the weeks. Walking cadence is maintained at home with a digital metronome or a free smartphone app. Patients included in this study were exposed to three follow-up visits (week 5 ± 1, T1; week 12 ± 1, T2; and week 20 ± 2, T3) to carry out an intermediate assessment in order to verify adherence and to update the exercise program. The number of training sessions, the walking time, and the number and duration of rest periods remained unchanged. Every patient was provided with a daily diary to report the completion of training and possible symptoms. Patients were also allowed to contact the rehabilitation team for any necessity, and during the first visit, they were familiarized with the home-based walking program. During the first visit (T0), a single session of exercise simulating the home-based training was performed by each patient under the supervision of the rehabilitation team to correct any possible mistakes during its execution. 

### 2.4. Outcomes Measures

Outcome measures were collected at baseline and during each intermediate visit by the same skilled assessor at all time points. A second healthcare professional not directly involved in the rehabilitation program collected all the data and set up the final dataset for the study.

#### 2.4.1. Infrared Thermography

The primary outcome in this observational analysis was the foot temperature measured by infrared thermal images. Thermal images were collected with a FLIRONE-Pro infrared camera (Flir System, Limbiate, Italy) connected to a smartphone with Android technology thanks to the FLIR One app. Every picture was taken with the patient in a supine position for almost ten minutes, and the following regions of interest (RoIs) were recorded: the anterior tibial artery, posterior tibial artery, arcuate pedis artery and dorsalis pedis artery [24]. The mean value of the four sampling points was also calculated. Temperature measurements of the selected RoIs were taken for both feet, and data were stored in the SD memory of the smartphone and downloaded to a computer to analyze each image with the FLIR Thermal Studio app for desktop. Thermal images were captured with the infrared camera perpendicular to the floor at a distance of 50 cm from the patient’s feet. Three consecutive thermal images were taken during each visit to ensure that all the RoIs were carefully represented.

#### 2.4.2. Secondary Outcomes

Secondary outcomes for this study included walking ability and hemodynamic assessment.

Walking ability was assessed by the 6 min walking test. The patient was asked to walk back and forth in a 20 m corridor, aiming to cover the maximal walking distance (six-minute walking distance, 6MWD). Patients were also asked to communicate the onset of claudication symptoms (pain-free walking distance, PFWD), and they were allowed to stop and restart after the necessary rest period.

Hemodynamic assessment included ankle brachial index calculation. The ankle brachial index was measured according to the published standard [25], with the patient lying down in the supine position; after 5 min of rest, using a Doppler ultrasound transducer (Dopplex SD2, Huntleigh Healthcare Ltd. Diagnostics, Cardiff, UK) and a standard blood pressure cuff, blood pressure was measured and recorded at both the posterior tibial arteries and dorsalis pedis arteries of both limbs. Systolic and diastolic blood pressure was also assessed in both arms.

### 2.5. Statistical Analysis

Data distribution was verified by a Shapiro–Wilk test. Baseline comparisons between sex and differences in foot perfusions were determined by independent samples *t*-test or Mann–Whitney tests. Overtime comparison of all variables was performed through a repeated-measures analysis of variance or a Freidman test according to data distribution. The variations between each time point were verified by paired-samples *t*-tests or Wilcoxon test as appropriate. Rank correlations between study variables were obtained with Spearman’s rho. The differences in response generated by sex were verified by a repeated-measures analysis of variance, with sex applied as a between-subject factor. A *p* value < 0.05 was considered significant. Statistical analyses were performed with MedCalc^®^ Statistical Software version 20.218 (MedCalc Software Ltd., Ostend, Belgium).

## 3. Results

**Figure 1:**
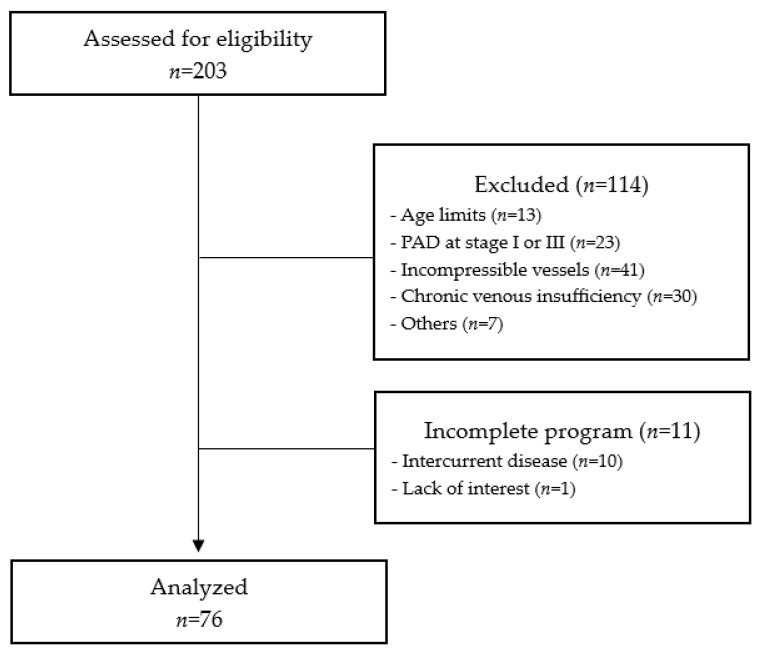
STROBE flow diagram of the study.

A total of 203 PAD patients were screened and enrolled in the rehabilitation program. For the purpose of this study, 114 patients were excluded for the reasons reported in Figure 1. Eleven patients did not complete the program for health reasons. 

A final sample of 76 patients was ultimately analyzed. The anthropometric and clinical characteristics of the population that completed the program are reported in Table 1.

All patients included in the analyses safely executed the exercise program without reporting any adverse events related to the training sessions. Patients executed a median of 85% of the prescribed training sessions.

At baseline, foot temperature was significantly lower in the limbs, with the worst vascular picture for all four districts examined (Table 2).

A correlation of low significance was observed between the baseline ABI and the mean temperature for both limbs (r = 0.20; *p* = 0.029). No other significant correlations between segmental pressures and baseline temperature in the respective RoIs were observed.

### 3.1. Overtime Variations

Significant overtime variations in temperature were observed for all the selected RoIs in both limbs. In particular, a mean increase of 1.3 °C was observed in the more diseased limb, with an mean increase of 0.8 °C in the contralateral limb. For the majority of the RoIs, significant variations (*p* < 0.05) were noted in the more diseased limb, even after 5 weeks of training. The data are presented in Table 3.

**Figure 2:**
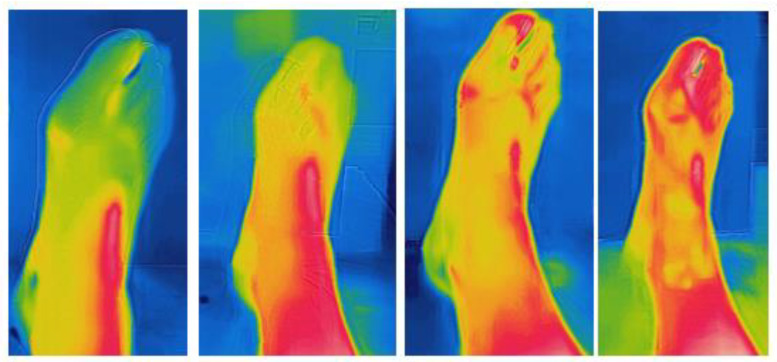
Overtime variations in foot temperature in a single patient across the four time points of collection.

A graphical representation of the foot of a single patient is presented in Figure 2.

### 3.2. Secondary Outcomes

The ABI values in both limbs showed a significant improvement (*p* < 0.001), with variations of 0.09 ± 0.07 in the more impaired limb and 0.06 ± 0.09 in the less impaired limb.

Significant variations were also observed for both functional outcomes, with a mean change of 123 ± 95 m (*p* < 0.001) for PFWD and 41 ± 52 m for 6MWD (*p* < 0.001).

### 3.3. Correlation between Outcome Variations

**Figure 3:**
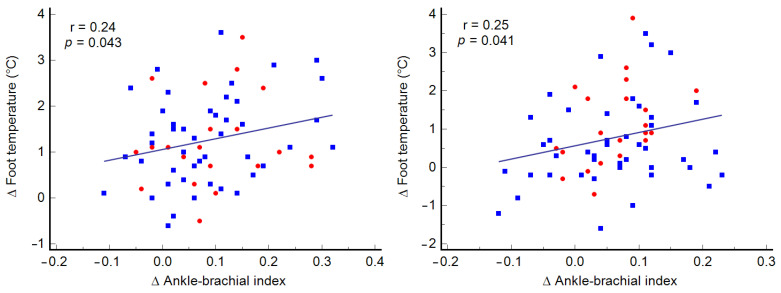
Rank correlation between variations in foot temperature and variations in ABI after the exercise program. Legend: blue squares, men; red circles, women.

A significant direct correlation was observed between changes in ABI value from baseline to the end of the program and variations in mean foot temperature for both the more diseased limb (r = 0.24; *p* = 0.043) and the less diseased limb (r = 0.25; *p* = 0.041). The data are shown in Figure 3.

No other significant correlations were observed between changes in foot temperature and performance.

### 3.4. Sex-Oriented Analysis

No differences in baseline demographics, comorbidities or PAD severity were observed according to sex (Supplementary Table S1).

**Figure 4:**
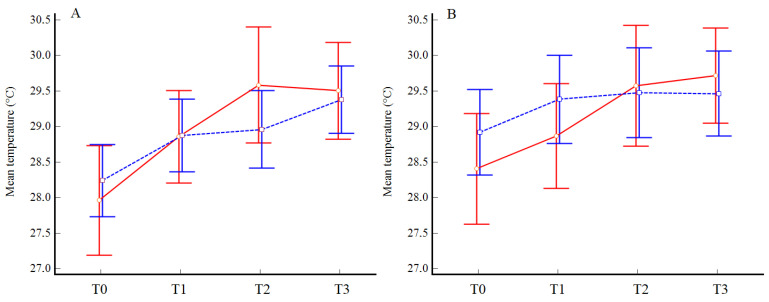
Overtime variations in mean foot temperature in the more impaired (**A**) and less impaired limb (**B**) in males (blue) and females (red).

The repeated-measures analysis of variance with sex employed as a between-subject factor did not highlight any significant difference in the mean temperature in the more diseased limb (*p* = 0.77) or in the contralateral limb (*p* = 0.70) Figure 4. The subsequent analyses including all the RoIs confirmed the absence of significant differences between the two subgroups (Supplementary Table S2).

## 4. Discussion

This observational study highlights concomitant temperature and perfusion-positive variations in both feet in PAD patients exposed to a low-intensity structured home-based program.

To the best of our knowledge, this is the first trial reported in the literature to employ IRT to monitor variations in foot perfusion after rehabilitation or exercise interventions. Several trials have evaluated foot perfusion after peripheral revascularization in PAD patients, with a range of variations from 0.5 °C to 3 °C [17,24,26,27,28,29]. In this observational study, a mean variation of +1.3 °C was observed in the more impaired limb, which is superimposable with values observed by Chang et al. after revascularizations in the limbs where ulcers healed [17] and significantly higher than those observed in other trials [26,28]. A considerable number of studies have used IRT for PAD diagnosis, for example, comparing the foot temperature of both limbs or employing an age- and sex-matched control group without PAD [16,29,30,31,32,33,34], while two papers report the effectiveness of pharmacological therapies in regulating foot temperature [35,36].

In the present study, we noted significant progressively increasing temperature variations at each time point of collection for most of the RoIs in both limbs without sex differences as other outcomes following the same home-based program [37,38]. These observed temperature changes are correlated with the congruent hemodynamic variations occurring in both feet, as measured by ABI, although with a lower coefficient with respect to the 0.7 value previously reported in a population without arterial calcifications [14].

The attainment of hemodynamic improvements in the foot is of critical importance for the management of PAD patients in order to favor walking ability and to prevent the transition toward more severe stages of the disease. Unfortunately, hemodynamic improvements after exercise training are not usually reported in PAD patients [39]. Conversely, the structured walking program proposed here was able to induce significant increases in lower limb perfusion. Walking training may induce nitric-oxide-mediated or hypoxia-induced effects able to contribute to vascular remodeling [40,41,42], but in particular, the low intensity of the training may represent the key factor favoring hemodynamic adaptations in the less perfused regions, considering the reported dose—response effect on vessels, especially after submaximal training [20,40,43,44,45]. In our study, the ubiquitous action of nitric-oxide-mediated variations were observed in both ABI and foot temperature in both limbs, as previously reported [20]. These changes are representative of the ability of exercise training to induce vasodilation in multiple arteries [46,47], unlike revascularization procedures, which are selective.

Interestingly, no differences were observed between sexes. In postmenopausal women, hormonal alterations may affect endothelial function and increase sympathetic activation [18]. These factors, together with the common presence of depression, may enhance vascular stress and contribute to small vessel disease, which is highly prevalent in women [18]. In this study, the type of exercise prescribed may have contributed to a similar positive response among sexes, considering that low- to moderate-intensity aerobic exercise has been found to be associated with improved angiogenetic stimulus and microvascular density, as well as lower sympathetic activation of the arteriolar tone [20,48,49]. To the best of our knowledge, changes in foot perfusion have not been measured previously in a sex-oriented analysis. Different outcomes following rehabilitation programs in chronic diseases have reported in women compared to men, related to interest/motivation, adherence to physical activity or effects achieved [50,51,52,53]. Considering the lower attitude of women toward exercise [50], this observation confirms the findings previously reported after the same home-based program [53] and the results of other authors who reported similar benefits in women compared to men after a low-intensity treadmill program [38,53,54,55]. To detect the changes occurring in foot perfusion, IRT may be a promising and useful method. This noncontact, noninvasive technology can selectively measure the temperature at precise points or angiosomes [56], and the operator can immediately see the exact value on the screen. Other methods adopted in the literature, such as NIRS or transcutaneous partial pressure of oxygen (TcPO2), have limitations with respect to the cost of the instruments, the time needed to set up the protocol and to analyze the data or the necessity to heat the zone under examination [11]. On the other hand, IRT has several issues still to be addressed, in particular, (i) the instrument characteristics and the measurement error, which is still high for the majority of available cameras; (ii) the measurement conditions (including room temperature and time of exposure), (iii) the RoI to be examined; and (iv) the intraindividual variation in skin temperature among individuals or the possible pathologies responsible for significant vasodilation or edema [13,16].

The limits of the procedure need to be included within the limitations of this observational study. In addition, we excluded patients with chronic venous insufficiency and patients with incompressible vessels because it was impossible to determine a relationship between temperature and ABI. The study design, particularly the lack of a control group and the lack of blinded assessors, may affect the external validity and generalizability of the data. Finally, a specific questionnaire was not developed to assess the variations in the perception of symptoms by the patients. 

## 5. Conclusions

A structured home-based walking program at low to moderate intensity was effective in improving foot perfusion in patients with PAD and intermittent claudication, without differences between sexes. Infrared thermography is a quick, reliable and easy-to-use method to monitor variations in foot temperature in almost every setting. Further studies including randomized controlled trials are needed to confirm the presented findings.

## Figures and Tables

**Table 1:** Characteristics of the subjects included in the study.

	Sample (*n* = 76)
Age (years)	74 ± 9
Male sex, *n* (%)	51 (67)
Smoking, *n* (%)	66 (87)
Hypertension, *n* (%)	69 (91)
Diabetes, *n* (%)	36 (48)
Hyperlipidemia, *n* (%)	52 (68)
Aortoiliac lesions, *n* (%)	18 (24)
Femoropopliteal lesion, *n* (%)	72 (95)
Tibial arteries lesion, *n* (%)	40 (53)
Charlson Comorbidity Index	6 ± 2
ABI, more impaired limb	0.59 ± 0.19
ABI, less impaired limb	0.86 ± 0.21
6 min walking distance (m)	298 ± 88
Pain-free walking distance (m)	145 ± 90

**Table 2:** Baseline values of ABI and foot temperature in both limbs in the whole population.

	More Impaired Limb	Less Impaired Limb	*p* Value
Ankle brachial index	0.63 (0.56–0.68)	0.88 (0.83–0.94)	<0.001
T mean (°C)	28.1 (27.7–28.4)	28.8 (28.3–29.3)	0.045
T anterior tibial (°C)	29.7 (29.2–30.2)	29.0 (28.5–29.5)	0.046
T posterior tibial (°C)	28.1 (27.7–28.7)	27.9 (27.5–28.4)	0.501
T dorsalis pedis (°C)	27.5 (27.1–28.0)	28.4 (27.8–28.8)	0.013
T arcuate pedis (°C)	28.0 (27.5–28.4)	28.7 (28.1–29.1)	0.061

**Table 3:** Variations in the outcome measures over time in the whole population.

RoIs of the More Impaired Limb	T0	T1	T2	T3	t for Trend *p* Value
Posterior tibial (°C)	27.9 27.5 to 28.4	28.6 * 28.1 to 29.1	28.8 * 28.3 to 29.3	29.2 * ‡ 28.8 to 29.6	6.33 <0.001
Anterior tibial (°C)	29.0 28.5 to 29.5	29.5 29.1 to 29.9	30.0 * 29.4 to 30.5	30.0 * 29.6 to 30.5	4.99 <0.001
Dorsalis pedis (°C)	27.5 27.1 to 28.0	28.3 * 27.9 to 28.8	28.7 * 28.2 to 29.1	28.8 * 28.4 to 29.3	6.74 <0.001
Arcuate pedis (°C)	28.0 27.6 to 28.4	29.0 * 28.6 to 29.4	29.2 * 28.8 to 29.7	29.6 * ‡ 29.1 to 30.1	7.67 <0.001
Mean (°C)	28.1 27.7 to 28.5	28.8 * 28.4 to 29.2	29.2 * 28.7 to 29.6	29.4 * ‡ 29.0 to 29.8	8.99 <0.001
RoIs of the Less Impaired Limb					
Posterior tibial (°C)	28.2 27.7 to 28.7	28.8 * 28.2 to 29.3	29.0 * 28.4 to 29.5	29.2 * 28.8 to 29.7	5.24 <0.001
Anterior tibial (°C)	29.7 29.2 to 30.2	30.2 29.7 to 30.7	30.4 * 29.9 to 30.9	30.3 29.8 to 30.7	2.70 0.008
Dorsalis pedis (°C)	28.4 27.9 to 28.9	28.8 28.2 to 29.3	29.0 * 28.5 to 29.6	29.2 * 28.6 to 29.7	4.22 0.001
Arcuate pedis (°C)	28.7 28.1 to 29.2	29.1 28.6 to 29.6	29.6 * 29.0 to 30.1	29.4 * 29.0 to 29.9	4.89 <0.001
Mean (°C)	28.7 28.3 to 29.2	29.2 * 28.7 to 29.7	29.5 * 29.0 to 30.0	29.5 * 29.1 to 30.0	5.35 <0.001
Hemodynamics					
ABI of the more impaired limb	0.59 0.54 to 0.63	0.67 * 0.61 to 0.73	0.67 * 0.62 to 0.72	0.68 * 0.62 to 0.73	5.16 <0.001
ABI of the less impaired limb	0.86 0.81 to 0.92	0.93 * 0.87 to 1.00	0.92 * 0.86 to 0.98	0.92 * 0.86 to 0.98	3.15 0.002
Performance					
Pain-free walking distance	145 125 to 166	208 * 185 to 231	249 * ‡ 224 to 273	268 * ‡ 242 to 293	11.76 <0.001
6 min walking distance	298 278 to 318	310 290 to 330	327 * ‡ 308 to 348	339 * ‡ § 319 to 359	7.34 <0.001

Legend: Data are expressed as means (95% confidence interval). * *p* < 0.05 with respect to baseline; ‡ *p* < 0.05 with respect to T1; § *p* < 0.05 with respect to T2.

## Data Availability

Data analyzed in this paper are available from the corresponding author upon reasonable request.

## Supplementary Materials

The following supporting information can be downloaded at: https://www.mdpi.com/article/10.3390/jpm13091312/s1, Table S1: Baseline characteristics of the two subgroups of subjects under study. Table S2: Values of Foot temperature over the ROIs in the more impaired limb in men versus women.
