# Supplementary material for: Foot Temperature by Infrared Thermography in Patients with Peripheral Artery Disease before and after Structured Home-Based Exercise: A Gender-Based Observational Study
Source: J Pers Med. 2023 Aug 27;13(9):1312. doi: 10.3390/jpm13091312 (PMC10532675; doi:10.3390/jpm13091312)
Supplement: Supplementary file 1 [file jpm-13-01312-s001.zip › jpm-2572316-supplementary.pdf]

Supplementary Table S1. Baseline characteristics of the two subgroups of subjects under study.

	Men (n=51)	Women (n=25)	p value
Age (years)	73 ± 7	75 ± 7	0.43
Smoking, n (%)	47 (92)	20 (80)	0.21
Hypertension, n (%)	47 (92)	22 (88)	0.56
Diabetes, n (%)	27 (52)	9 (36)	0.16
Hyperlipidemia, n (%)	36 (70)	16 (74)	0.56
Charlson Comorbidity Index	6 ± 2	6 ± 2	0.75
ABI, more impaired limb	0.58 ± 0.20	0.61 ± 0.17	0.44
ABI, less impaired limb	0.86 ± 0.22	0.85 ± 0.20	0.69
6-minute walking distance (m)	309 ± 93	275 ± 70	0.12
Pain-free walking distance (m)	154 ± 88	127 ± 93	0.21

Supplementary Table S2. Values of Foot temperature over the ROIs in the more impaired limb in men versus women. * $p < 0.05$ with respect to baseline; ‡ $p < 0.05$ with respect to T1.

ROIs more impaired limb MEN	T0	T1	T2	T3	t for trend P value
Posterior tibial (°C)	28.2<br>27.7 to 28.7	28.7<br>28.1 to 29.3	28.7<br>28.0 to 29.3	29.4*<br>28.9 to 29.8	4.79<br><0.001
Anterior tibial (°C)	28.9<br>28.3 to 29.6	29.4<br>28.8 to 29.9	29.6*<br>29.0 to 30.2	29.8<br>29.2 to 30.3	3.45<br>0.011
Dorsalis pedis (°C)	27.7<br>27.1 to 28.2	28.4*<br>27.8 to 29.0	28.5*‡<br>27.9 to 29.0	28.9*‡<br>28.4 to 29.5	5.48<br><0.001
Arcuate pedis (°C)	28.1<br>27.6 to 28.6	29.0*<br>28.4 to 29.5	29.1*<br>28.5 to 29.7	29.4*<br>28.9 to 29.9	5.78<br><0.001
Mean (°C)	28.2<br>27.7 to 28.7	28.9*<br>28.4 to 29.4	29.0*<br>28.4 to 29.5	29.4*<br>28.9 to 29.9	6.96<br><0.001
ROIs less impaired limb WOMEN	T0	T1	T2	T3	t for trend P value
Posterior tibial (°C)	27,8<br>26,5 to 28,3	28,3*<br>27,4 to 29,2	29,0*<br>28,1 to 29,9	28,8<br>28,0 to 29,6	4.02<br><0.001
Anterior tibial (°C)	29,2<br>28,4 to 30,1	29,9<br>29,3 to 30,4	30,6*<br>29,7 to 31,6	30,6*<br>29,9 to 31,2	3.72<br>0.001
Dorsalis pedis (°C)	27,3<br>26,4 to 28,2	28,1<br>27,3 to 28,9	29,0*<br>28,2 to 29,9	28,6<br>27,6 to 29,6	3.81<br><0.001
Arcuate pedis (°C)	27,9<br>27,1 to 28,7	29,0*<br>28,3 to 29,7	29,6*<br>28,8 to 30,5	30,0*<br>29,0 to 30,9	5.10<br><0.001
Mean (°C)	28.0*<br>27,2 to 28,7	28,9*<br>28,2 to 29,5	29,6*<br>28,8 to 30,4	29.5*<br>28,8 to 30,2	5.75<br><0.001
